# Non-adjacent visual dependency learning in chimpanzees

**DOI:** 10.1007/s10071-015-0840-x

**Published:** 2015-01-21

**Authors:** Ruth Sonnweber, Andrea Ravignani, W. Tecumseh Fitch

**Affiliations:** 1 Department of Cognitive Biology, University of Vienna, Althanstrasse 14, 1090 Vienna, Austria; 2 Language Evolution and Computation Research Unit, School of Philosophy, Psychology and Language Sciences, University of Edinburgh, 3 Charles Street, Edinburgh, EH8 9AD UK

**Keywords:** Feature based, Arbitrary associative, Operant task, Touch screen, Non-human primates

## Abstract

Humans have a strong proclivity for structuring and patterning stimuli: Whether in space or time, we tend to mentally order stimuli in our environment and organize them into units with specific types of relationships. A crucial prerequisite for such organization is the cognitive ability to discern and process regularities among multiple stimuli. To investigate the evolutionary roots of this cognitive capacity, we tested chimpanzees—which, along with bonobos, are our closest living relatives—for simple, variable distance dependency processing in visual patterns. We trained chimpanzees to identify pairs of shapes either linked by an arbitrary learned association (*arbitrary associative dependency*) or a shared feature (same shape, *feature*-*based dependency*), and to recognize strings where items related to either of these ways occupied the first (leftmost) and the last (rightmost) item of the stimulus. We then probed the degree to which subjects generalized this pattern to new colors, shapes, and numbers of interspersed items. We found that chimpanzees can learn and generalize both types of dependency rules, indicating that the ability to encode both feature-based and arbitrary associative regularities over variable distances in the visual domain is not a human prerogative. Our results strongly suggest that these core components of human structural processing were already present in our last common ancestor with chimpanzees.

**Electronic supplementary material:**

The online version of this article (doi:10.1007/s10071-015-0840-x) contains supplementary material, which is available to authorized users.

## Introduction

Humans have a strong tendency to mentally arrange their perceptual worlds into structured elements and sequences and to organize their surroundings into patterns. This is particularly evident when looking at natural languages, music, or visual patterns humans produce (e.g., Fitch 2006; Westphal-Fitch et al. 2012). Statistical or rule-based strategies are applied to learn and detect such regular structures (Pena et al. 2002; Perruchet et al. 2004), and the ability to extract rules from perceptual stimuli is present early in human infants (Aslin et al. 1998; Kirkham et al. 2002; Marcus et al. 1999; Saffran et al. 1996).

Structural regularities are also present in many animal species’ own communication systems, and a recent review by ten Cate and Okanoya summarizing the main findings from non-human animal patterning experiments concluded that several non-human species possess basic rule learning abilities (ten Cate and Okanoya 2012). However, such abilities remain unexplored in many species.

A crucial requirement for processing regularities (e.g., in speech streams) is the ability to perceive and represent relationships between elements separated in space and time (Gebhart et al. 2009; Newport and Aslin 2004; van Heugten and Shi 2010). A multitude of such non-adjacent dependency structures is found in natural languages, at both word and morphemic levels (van Heugten and Shi 2010). A standard morphosyntactic example for non-adjacent regularities is “agreement”, for example, the matching of auxiliary verb (e.g., is) and main verb endings (e.g., -ing) in English (Santelmann and Jusczyk 1998). Humans can also track relationships between non-adjacent, perceptually similar elements in artificial languages (Newport and Aslin 2004).

**Fig. 1:**
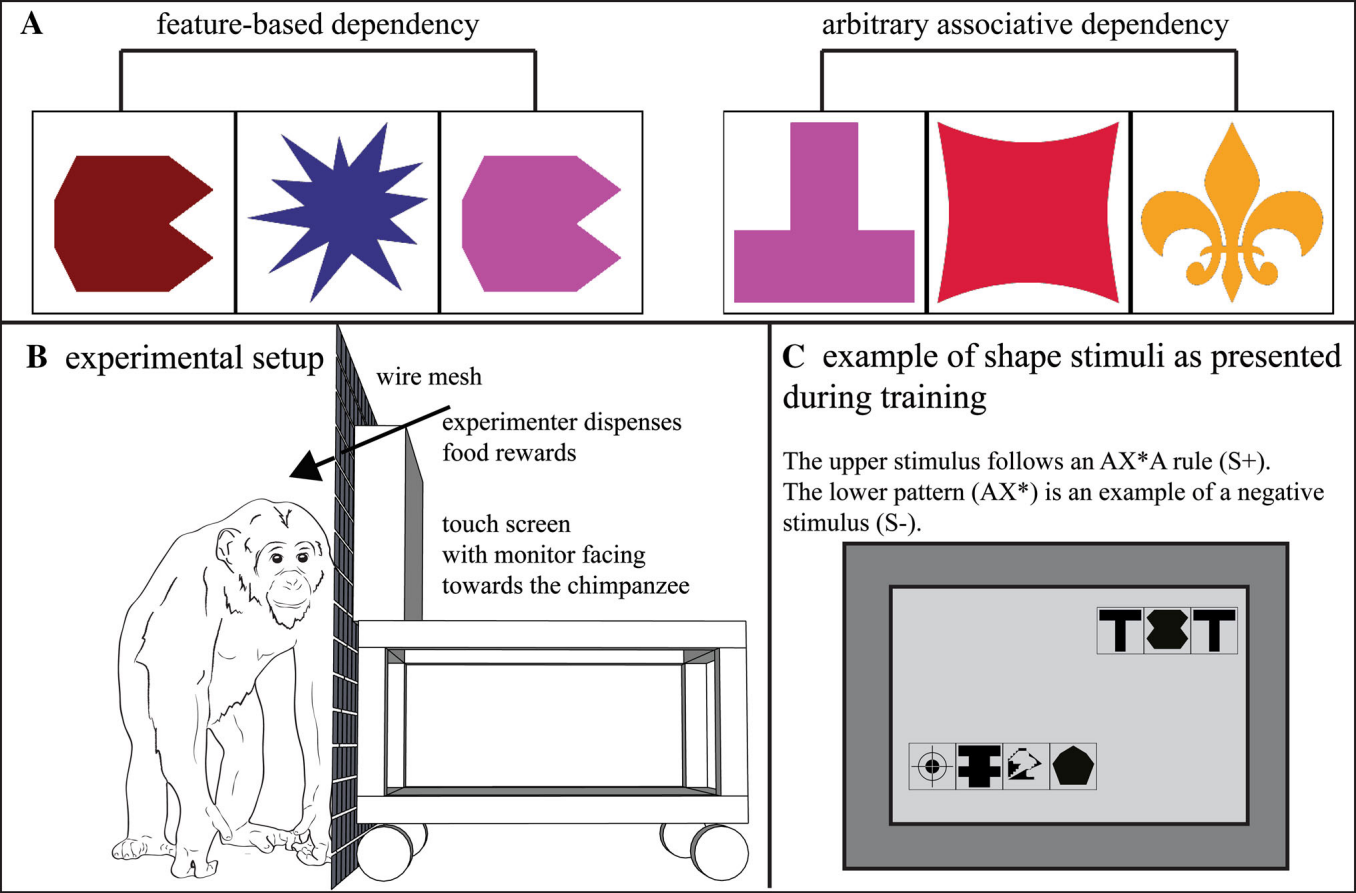
**a** Examples of feature based and arbitrary associative dependencies in artificial visual patterns. In feature-based dependencies (*left*), two adjacent or non-adjacent elements share a common feature (here shape). Dependent elements are not identical (here they may differ in *color*), but belong to identical classes (any shape, but the same shapes for both dependent elements). Arbitrary associative regularities (*right*) have dependencies between a priori unrelated elements. **b** Touch screen setup. The setup was mounted on a table with wheels to allow for flexibility in the testing location. The setup consisted of a touch-sensitive monitor (facing toward the experimental subject), an experimenter monitor (facing toward the experimenter), a Mini Mac, a keyboard, and an optical mouse. Chimpanzees could reach through a wire mesh with their fingers to touch stimuli on the screen. The experimenter dispensed pieces of high quality food rewards with a tong for correct choices in training trials. **c** Examples of training stimuli. Training stimuli consisted of a series of abstract black-and-white geometrical shapes (2–4, each with a square black frame) arrayed in a horizontal row. Two stimuli were presented simultaneously (a positive, S+ stimulus and a negative, S− stimulus)

Relationships, or dependencies, may be either *feature*-*based* or based on *arbitrary associations* (for examples see Fig. 1). Recognizing that two adjacent or non-adjacent elements belong to the same perceptual or logical category, based on one or more shared features, is necessary for feature-based dependency encoding (Newport and Aslin 2004). Dependent elements are not identical, but share traits that belong to the same class (e.g., same shapes or same colors, cf. Fig. 1a). Such representations are abstract in that they allow generalization to unfamiliar, novel perceptual items. On the other hand, establishing associative relationships between a priori unrelated elements is crucial to detecting arbitrary associative regularities (Toro and Trobalon 2005). Some combination of specific elements must be learned, and a certain relationship between them represented (e.g., A always precedes B, Fig. 1a). Because this type of dependency is encoded on a less abstract level, less flexible computations are possible (i.e., no inferences about C and D can be made from the learned association between A and B). However, if the position of first items (A and C) and last items (B and D) of several learned associative pairs, as well as the relation between them, are represented on a more abstract level, computations recombining elements will be possible (i.e., A precedes D and C precedes B).

In addition, matched elements can exhibit positional regularities. Not only do dependent items and their relation to each other have to be represented mentally in such a case, but information about their (absolute and/or relative) position in relation to other elements in a sequence must be encoded as well. For example, two items may be adjacent or only present at the edges, that is, items that are linked by a dependency occurring at the beginning and end of the string (Endress et al. 2010).

Previous studies demonstrate dependency sensitivity in various animal species in the acoustic domain: van Heijningen and colleagues (van Heijningen et al. 2009, 2013) demonstrated for zebra finches that simple adjacent dependency rules (same or different elements) can explain results from complex pattern processing experiments. Cotton-top tamarins (Newport et al. 2004) and rats (Murphy et al. 2008) can successfully learn abstract dependencies between elements at fixed distances, while chimpanzees can process dependencies between specific items at variable distances in the acoustic domain (Endress et al. 2010). In an auditory task similar to the one used here, squirrel monkeys proved capable of spontaneously detecting (without training) abstract, non-adjacent dependencies between elements in acoustic strings and generalizing to novel classes and to new stimulus lengths (Ravignani et al. 2013b).

Abstraction, rule formation, and rule application across different tasks have been argued to contribute to the high flexibility underlying human intelligence (Emery and Clayton 2004). Because chimpanzees (along with bonobos) are our closest living relatives, they are a key species for understanding the roots of human cognitive abilities such as the ability to detect particular types of dependencies. Many previous studies employed habituation–discrimination paradigms (Endress et al. 2010; Newport et al. 2004; Ravignani et al. 2013b), which provide insights into *spontaneous* detection of sensory dependencies. However, spontaneous capabilities do not necessarily indicate the cognitive limitations present in a particular species: Animals may notice changes in presented stimuli but fail to respond with an observable behavioral change, leading to “false negative” results. A clear demonstration of strong limitations typically requires considerable training, for example, using operant techniques (ten Cate and Okanoya 2012). The reward system applied in operant tasks keeps the animals motivated to respond to all perceived changes in presented stimuli. Furthermore, operant tasks allow for a more fine-grained battery of test stimuli than is possible with habituation/discrimination methods.

In this study, we tested chimpanzees’ ability to learn variable distance dependency rules in visual patterns in an operant task and then explored their ability to generalize such rules to previously unseen stimuli. Chimpanzees were trained to detect dependencies between elements at the edges of visual stimuli of different lengths. These dependencies could either be feature based (henceforth: AA group) or based on arbitrarily associated item pairs (AB group). The purpose of testing feature-based dependencies (patterns following an AX*A rule, where A’s are items sharing a particular feature, X indicates a nontarget object, and * a variable number of items) was to establish whether chimpanzees can represent relational categories between elements located at a distance and process the dependency relationship between them (Abe and Watanabe 2011; Ravignani et al. 2013b). The purpose of testing arbitrary associative dependencies (with patterns following an AX^*^B rule, where As and Bs were previously trained associative pairs) was to ascertain whether chimpanzees can, once exposed to specific pairs of items (which are linked by an arbitrary learned association, rather than physical features of the stimulus), generalize this relation across variable numbers of intervening items, and to previously unseen combinations of elements (Rey et al. 2012). Furthermore, we wanted to understand to what extent chimpanzees encode positional relations between dependent elements in visual patterns (e.g., recognizing that dependent elements are always located at the edges of visual patterns).

In principle, the necessary mental representations underlying feature-based and arbitrary associative dependencies should differ in the degree of abstraction and consequently in the flexibility of generalizations they allow. We therefore hypothesized that mental representations of dependencies between elements that share a common feature, assigning them to the same category (feature-based), would allow flexible generalizations. Since chimpanzees are relatively proficient in categorization tasks (Spinozzi 1993, 1996; Tanaka 1995), while some monkeys have been shown to be sensitive to abstract dependencies (Ravignani et al. 2013b), we predicted that chimpanzees could learn a feature-based dependency rule and would be capable of generalizing this rule to novel stimuli.

Establishing arbitrary associative relationships between specific pairs of items, in contrast to feature-based dependencies, demands little abstraction. Previous studies with chimpanzees demonstrated that, at least in the auditory domain, regularities with target elements at the edges of strings are particularly easy to detect (Endress et al. 2010). This suggested that our study subjects would also be able to successfully encode positional relation between dependent items and their relation to each other in abstract visual stimuli. We thus predicted that chimpanzees would be capable of detecting arbitrary associative dependencies involving trained pairs of elements, independently of stimulus length and amount of distracting information.

Finally, more flexible computations with arbitrary associative dependencies would be possible if relations between classes of items (e.g., “beginning” and “ending” items, assuming reading from left to right) were encoded at a more abstract level than necessary for simply representing specific single item pair associations. Thus, we also tested if our chimpanzee subjects could flexibly recombine beginning and ending elements across associative pairs. We hypothesized that if representations of this spatial dependency rule were encoded on an abstract level, chimpanzees should be able to perform more flexible computations and thus generalize arbitrary associative regularities across trained item pairs (e.g., when trained on AXB and CXD, they should accept AXD but reject DXA stimuli).

## Methods

### Animal welfare and ethics

The study was approved by the scientific board of the Living Links and Budongo Research Consortium (Royal Zoological Society Scotland). All experimental procedures were in accordance with British, Austrian, and European Union law. No invasive methodologies were applied at any point of the study: There was no food or water deprivation, and only positive reinforcement techniques were used for chimpanzee training. Individuals who participated in this study did so on a strictly voluntary basis, in return for food reward, and could leave the experiment at any time.

### Study site and study subjects

Research was conducted at the *Budongo Trail* facility at Edinburgh Zoo, Scotland, UK. At the beginning of this study, this socially housed group of chimpanzees encompassed 19 individuals: 11 females and 8 males between 14 and 49 years of age. The chimpanzees were housed in three interconnected indoor enclosures (pods of 12 × 12 × 14 m each) and an outdoor enclosure (1,832 m^2^), which was accessible to the chimpanzees during the day (Ravignani et al. 2013a). The design of the facility allowed the group to split into subgroups or join into one group, exhibiting the natural fission–fusion behavior of this species. Water was available ad libitum, and food was provided four to five times a day.

Fourteen of the 19 resident chimpanzees had previously been trained to use a touch screen (Herrelko 2011) and perform a simple two-alternative forced-choice (2AFC) task (choosing a red over a green circle of equal size). Eleven of these 14 touch screen-skilled individuals participated on a regular basis and received training for the experiments reported here (detailed description below).

### Experimental setup and procedure

Chimpanzees gave responses using a touch-sensitive screen (15 in. Elo Touch Systems, Carroll Touch Technology), connected to an Apple Mini Mac computer (Fig. 1b). An additional computer monitor (for the experimenter), a keyboard, an optical mouse, and a set of loudspeakers providing acoustic feedback for correct and incorrect choices (one sound assigned to correct answers and another to incorrect answers), were also connected to this computer. The setup was mounted on a rolling table, allowing testing in all compartments of the chimpanzee testing area (for a detailed description see supplementary material Annex A). Custom-written Python code (www.python.org) was used to generate stimuli, control experiments and log the data.

The chimpanzees were trained and tested using a 2AFC task (Fig. 1c). Each training and testing session consisted of 12 trials, and within one research session, a maximum of four sessions per individual was possible (48 trials). In order to move to the next training stage, or proceed to testing, a chimpanzee had to make 33 first correct choices within a total of 48 trials (power analysis performed using a binomial distribution, *P* < 0.001). Positive reinforcement was used for training: Correct choices elicited an acoustic secondary reinforcement signal (a clicker sound familiar to all chimpanzees from previous husbandry training), and the experimenter dispensed a highly preferred food reward with tongs (depending on individual and day: grapes, blueberries, peanuts, date pieces, or raisins). After wrong answers in training trials, an unappealing acoustic signal (short irregular series of non-pulsatile sounds) was played, and a red penalty screen displayed for 3 s; failed training trials were repeated immediately until the individual chose the correct stimulus.

Because previous work in visual artificial grammar learning (AGL) experiments suggests that staged input training can promote learning performance (Conway et al. 2003), our study subjects were trained in stages of gradually increasing difficulty (for a detailed description of training stages see supplementary material Annex B). Six individuals were trained for *feature*-*based dependencies* (“AA group”), while five chimpanzees received training for *arbitrary associative dependencies* (“AB group”) in a series of training steps. Stimuli were randomly presented at four possible positions of the monitor (upper and lower right and left corners) to discourage side or location biases. By touching anywhere within either of these stimuli, the individual registered its choice and (in training trials only) received feedback.

**Fig. 2:**
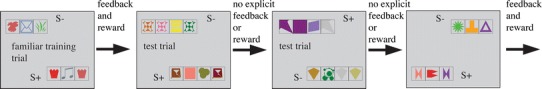
Example sequence of trials as presented during test sessions. A familiar repetition trial (showing one S+ and one S− stimulus) was presented. A sound signal provided feedback after a choice, and a penalty screen was shown after wrong choices. After correct choices a food reward was provided. Then, a test trial with one S+ and one S− stimulus was presented. Choices in test trials did not trigger feedback and were not rewarded. Then, another test trial (never more than two in a row) or a familiar repetition trial was shown randomly. A total of six test trials were interspersed with familiar repetition trials

During subsequent test sessions, six rewarded trials familiar from training (“repetition trials”) with contingent rewards were interspersed with six novels “test trials” lacking any explicit differential feedback (Fig. 2). Rewarded repetition trials were presented during test sessions to prevent decreased motivation and consequent non-participation (since 50 % of all trials were still potentially rewarded). These trials also provided a measure of our subjects’ attentiveness during any particular test session. Only when most of the previously trained trials were correctly answered (*P* < 0.05) were responses to test trials considered representative of the chimpanzee’s potential performance (see Results). This criterion was specified before any analysis commenced and applied to all test trials. To investigate whether this method of session validation was justified, we tested whether performance in familiar repetition trials predicted performance in test trials, based on percentages of correct choices per session. Percentages of correct choices in familiar repetition trials were positively correlated with performance in test trials (*r* = 0.208, *df* = 291, *P* < 0.001).

### Stimuli

**Fig. 3:**
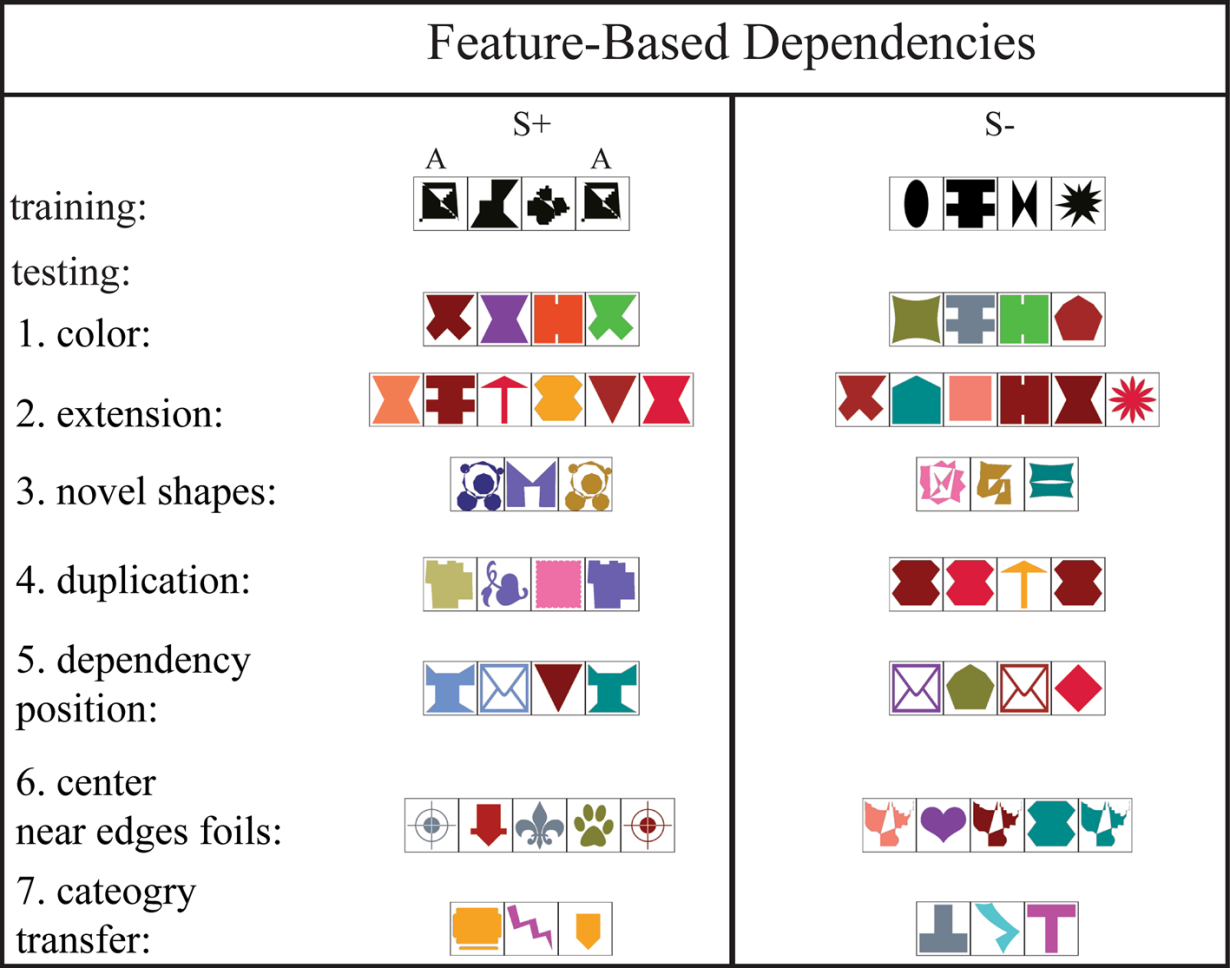
Training and test stimuli for the AA group (feature based). Training stimuli always consisted of a series of black shapes, where elements in S+ were arrayed following an AX^*n*^A pattern (*n* = 1 or 2) and S− strings followed an AX^*n*+1^ (*n* = 1 or 2) rule. This was maintained for Test 1 (*color test*), but the shapes composing the strings were colored. The dependency distance was varied by increasing the number of Xs in Test 2 (extension test) (S+: AX^*n*^A and S−: AX^*n*+1^, where *n* = 3, 4, or 5). For Test 3 (novel shapes test), stimulus length was reduced (S+: AX^*n*^A and S−: AX^*n*+1^, where *n* = 1 or 2), but entirely unfamiliar shapes and colors were used for stimulus design. Test 4 (duplication foils) tested AX^*n*^A (S+) against AAX^*n*−1^A, where *n* = 2 or 4. To test absolute and relative dependency position (Test 5), chimpanzees were presented with S+ following AX^n^A and S− following AX^*n*−1^AX^*n*−1^, or X^*n*−1^AX^*n*−1^A, where *n* = 2 or 4. Foils with additional recurrences of dependent elements in the center or near the edges of visual strings were presented in Test 6 (center foils: S+: AX^*n*^A, where *n* = 3 or 5 and S−: AX^*n*^AX^*n*^A, where *n* = 1 or 2; near edges foils: S+: AX^*n*^A, where *n* = 4 or 5 and S−: AX^*m*^AX^*n*^A or AX^*n*^AX^*m*^A, where *n* = 3 and *m* = 1). Test 7 followed the same patterns as Test 1, but colors (not shapes) had to be matched

**Fig. 4:**
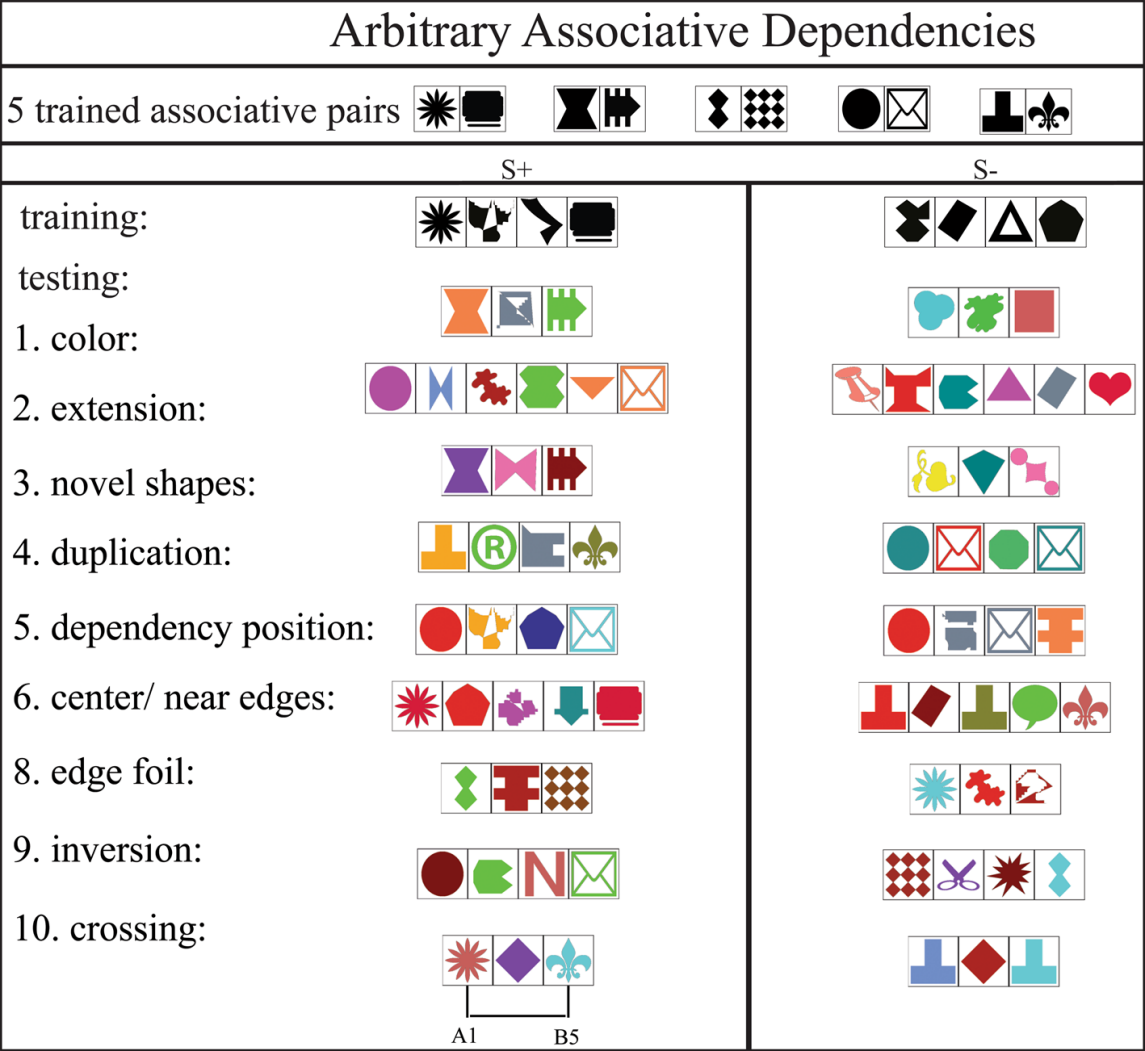
Training and test stimuli for the AB group (arbitrary association). Training stimuli consisted of a series of black shapes, where A_*i*_ and B_*i*_ were associated by learning and separated by one or two X elements [S+ followed an AX^*n*^B pattern (*n* = 1 or 2) and S− strings an X^*n*+2^ (*n* = 1 or 2) rule]. The same configurations were used in Test 1 (color test), but the individual shapes were colored. To test for increased dependency distances, more Xs were introduced in between associated AB pairs (S+: AX^*n*^B and S−: X^*n*+2^, where *n* = 3, 4, or 5) in Test 2 (extension test). For Test 3 (novel shapes test), S+ followed AX^*n*^B and S− X^*n*+2^, where *n* = 1 or 2 and stimuli consisted of novel shapes and colors. In Test 4, AX^*n*^B (S+) and four variants of S− (AAX^*n*−1^B, AX^*n*−1^BB, ABX^*n*−1^B or AX^*n*−1^AB, where *n* = 2 or 4) were used. Absolute and relative dependency position were tested with S+ following AX^*n*^B and S− following either AX^*n*−1^BX^*n*−1^ or X^*n*−1^AX^*n*−1^B, where *n* = 2 or 4. Foils with recurrences of dependent elements in the center or near the edges of the visual patterns were presented in Test 6 (center foils: S+: AX^*n*^B, where *n* = 3 or 5 and S−: AX^*n*^BX^*n*^B or AX^*n*^AX^*n*^B, where *n* = 1 or 2; near edges foils: S+: AX^*n*^B, where *n* = 4 or 5 and S−: AX^*m*^AX^*n*^B, AX^*n*^BX^*m*^B, AX^*n*^AX^*m*^B or AX^*m*^BX^*n*^B where *n* = 3 and *m* = 1). Test 8 (Test 7 only for the AA group) presented S+ (AX^*n*^B, where *n* = 1 or 3) with foils missing the first or the last dependency element (X^*n*+1^B, AX^*n*+1^, where *n* = 1 or 3). In the inversion test (Test 9), positions of As and Bs were swapped (S+: AX^*n*^B and S−: BX^*n*^A, where *n* = 1 or 2). Finally, in Test 10, As and Bs of different associative pairs were scrambled (S+ A_*i*_X^*n*^B_*j*_ and S− A_*i*_X^*n*^A_*i*_ or B_*i*_X^*n*^B_*i*_, where *n* = 1, 2 or 3)

Visual stimuli consisted of a series of square, framed individual elements arrayed in a horizontal row. Single elements incorporated many different abstract geometrical shapes, surrounded by a square black frame (outer dimensions 225 × 225 pixels). Black shapes were used as elements for training stimuli, while during testing, shapes were differently colored (testing shapes were never black, and all shapes could, but did not necessarily, differ in color). To construct positive (S+) and negative (S−) stimuli (Figs. 3, 4), between two and seven elements were linearly concatenated. In the following description of the visual patterns, we use capital letters A and B to denote target elements within strings. Recurrences of those (e.g., AA) indicate two elements with identical shapes (but different colors during testing). When two different elements, say A and B, become associated by training, we use a lower common index to refer to this arbitrary learned association (e.g., A_1_ B_1_ denotes two associated but different shapes). Xs stand for nontarget distractor elements, which are not members of the categories above. Unlike As and Bs, recurrence of distractors (e.g., XX) stands for two elements of different shape. Finally, the exponent is used to indicate recurrences, e.g., X^2^ = XX and AX^3^A = AXXXA, while a star AX*A indicates a variable number of X elements. Stimuli used in training varied only in shape (black shapes in frames of identical outer dimensions).

#### Feature-based dependency (AA) group

In the AA group, the positive stimuli followed an AX*A pattern, where the dependencies between elements involved the abstract feature of shape: A elements could be any possible shape, as long as it was the same for both A elements (regardless of color). Hence, the shape of the first and last element of the positive stimulus was identical, defining a “same shape” relation independent of any particular shape (see Fig. 3). The pool of shapes from which elements were sampled encompassed 70 different shapes, which were used for stimulus creation for all training phases. By sampling from this relatively large pool of elements, we tried to encourage individuals to learn the abstract relationship, rather than to memorize specific shape configurations. A and X shapes for the AX*A pattern were sampled from the same pool of shapes. Any shape could occur as either an A or X element in different stimuli. However, within a stimulus, if a shape was used as an A element, it was never used as an X element (and vice versa). Training stimuli were maximally four elements long (AXA, AX^2^A as S+ and AX^2^, AX^3^ as S−). The same pool of shapes was used during testing, where all 70 shapes were presented, but now in one of 21 different non-black colors. Here, dependent elements still shared the same feature “shape”, but could differ in color. Thirty additional shapes in 15 different colors (not black) were introduced in Test 3 (for a description of test stimuli and test types see Fig. 3).

#### Arbitrary associative dependency (AB) group

In the AB group, five chimpanzees had to learn to associate five specific pairs of different shapes with each other (A_1_B_1_, A_2_B_2_, A_3_B_3_, A_4_B_4_, A_5_B_5_). Unlike AA stimuli, these pairs of shapes had neither any obvious a priori connections nor non-random shared features with each other (see Fig. 4). Instead, we trained the chimpanzees on specific associations between arbitrary pairs (denoted here with the same numeric index), involving arbitrary associative dependencies. Elements used to define A and B dependency could *not* occur as distractor X shapes (and vice versa). Hence, clear categories for distractor shapes (a pool of 60 different shapes and 30 additional shapes from Test 3 onwards) and associative pairs of target shapes (10 different shapes) were created. The same pool of stimuli was used for all subjects. Again, no color was used in training stimuli—all shapes were black, and training stimuli did not exceed a length of four elements (AXB, AX^2^B as S+ and X^3^, X^4^ as S−). Specific A elements were always displayed with corresponding B elements (A_1_ with B_1_, A_2_ with B_2_, etc.). No scrambles, such as A_1_ with B_2_, occurred during training. Again, during testing the dependent and the distractor elements were colored (21 colors for the first shape pool and 15 colors for shapes in the second shape pool, all non-black; colors were assigned randomly to individual shapes), and colors of the two dependent elements could differ from each other (for further details see Fig. 4).

### Test types

Both groups (AA and AB group) were tested with a series of “generalization” tests (see Figs. 3, 4 for visual representations and stimulus descriptions: Test 1–3 for both groups, Test 7 for the AA group, and Test 9 and 10 for the AB group). The aim was to present our study subjects with stimuli containing the dependencies from training, but also possessing some novel features (such as color, distance between dependent elements, etc.) over which the subjects had to generalize the learned rule. Both positive and negative stimuli were coupled in a way that depended on the generalization type (i.e., when stimulus length was increased to test for generalization over dependency distance, the number of elements in the stimuli was increased by the same number in positive and negative stimuli).

In another set of tests (“foil tests, see Fig. 4 for visual representations and stimulus descriptions: Test 4, 5, and 6 for both groups as well as Test 8 for the AB group) a stimulus containing the dependency was presented with a foil pattern altering or lacking the dependency in a specific way. These tests allowed us to test for specific strategies individuals used to solve the task and to draw conclusions concerning what rule (if any) they had learned.

In Test 1, we presented subjects with colored stimuli (familiar rewarded repetition trials showed black stimuli). As in either group’s last training step, visual patterns consisted of three or four elements, but unlike in the training, test stimuli were colored. Colors were assigned randomly to individual elements such that all shapes could have the same or different colors. This first test probed chimpanzees’ ability to ignore irrelevant, distracting color information. After Test 1, colored stimuli (instead of black stimuli) were used for subsequent familiar repetition trials so that chimpanzees could not easily distinguish between novel, unrewarded, and not fed-back test trials (colored elements) and rewarded, fed-back repetition trials. In Test 2, we increased the length of the visual patterns by introducing more distractor elements. Strings now contained five, six, or seven elements (including the dependent elements). This probed chimpanzees’ ability to generalize across variable dependency distances. In Test 3, visual patterns were constructed with novel, unfamiliar shapes (30 different shapes in 15 different colors) for the AA group. These novel unfamiliar shapes were also used for distractor elements in the AB group patterns, while elements of the associative pairs remained the same. Hence, this test is particularly relevant for the AA group. While the AB group had to generalize over different distractor shapes, individuals of the AA group had to additionally generalize to dependency elements with novel shapes, thus broadening their “same” relation to include previously unseen shapes.

After these three generalization tests, we confronted the study subjects with a set of three different foil tests. None of the foils presented during these tests violated the dependency rule, but altered it in some way. S− stimuli in Test 4 showed additional recurrences of a dependency element either at the beginning or at the end of the string (thus S− exhibited an AAX*A, AX*AA, or AAX*B and AX*BB pattern, respectively). These additional recurrences were consistently located at the edges of the stimuli. In Test 5, we tested for the processing of the position of the target items: We presented stimuli containing the relative, but not the absolute positional relation between dependent elements. Foil strings following X*AX*A, AX*AX* or X*AX*B and AX*BX* patterns were presented with S+ stimuli displaying the usual dependency elements at the edges. Finally, in Test 6, strings with additional recurrence of a dependent element near the edges (AX^n^AX^m^A, AX^m^AX^n^A, and AX^n^AX^m^B, AX^m^BX^n^B, respectively) or in the center of the patterns (AX^n^AX^n^A, AX^n^AX^n^A, and AX^n^AX^n^B, AX^n^BX^n^B, respectively) were displayed. Thus, Test 4 and Test 6 examined whether chimpanzees differentiated patterns containing the (binary) trained dependency relation only from foils with additional recurrence of the dependent elements at different positions: at the edges, near the edges, and at the center of a string. Earlier experiments demonstrated that edges of acoustic strings can function as anchor points when processing positional regularities in acoustic sequences (Endress et al. 2010) suggesting that chimpanzees may be more likely to reject foils with recurrences at the edges of visual patterns.

The final test for the AA group (Test 7) examined whether chimpanzees would spontaneously over-generalize the dependency rule from shapes to colors as category classes. While all training and testing steps showed shape class-dependent elements, and color represented an irrelevant variation within this category, Test 7 reversed this relationship: Individuals had to spontaneously match color and ignore variation in shape. This was a very ambitious test, as success would require a particularly high level of flexible abstraction of “same feature”, going beyond the previous test stimuli. Furthermore, it demanded an inversion between the formerly irrelevant distractor feature and the shape feature previously relevant for categorization.

The AB group was presented with three further tests. Test 8 was another foil test. Either the A at the beginning or the B at the end was missing in foil stimuli (S−). This test was designed to allow inferences concerning the amount of attention individuals paid to the matched pair when at the edges of the stimuli. The two final tests were particularly relevant for language-relevant interpretations. In the probe stimuli in Test 9, the positions of As and Bs were inverted (assuming a left to right parsing direction: A’s now at the end, and B’s at the beginning of strings). Discriminating these patterns from strings in the trained configuration (normal: A’s at the beginning and B’s at the end) would imply that individuals had constructed a positional, sequential relationship between the two categories, namely A’s must occur to the left of any B. Finally, in Test 10, the chimpanzees’ ability to process novel combinations of dependent item pairs (that is A_*i*_X*B_*j*_, where *i* ≠ *j*) was probed (cf. Rey et al. 2012). Corresponding S− stimuli followed an A_*i*_X*A_*i*_ or B_*i*_X*B_*i*_ pattern (with shapes in different colors, see Fig. 3). Accepting shuffled A–B combinations would imply that chimpanzees had formed categories of A elements (“starting” or “left”) and B elements (“ending” or “right”) and could flexibly combine members of these categories to accept novel AB configurations.

### Statistical analysis

Choices were analyzed for each individual, considering each test separately. One-tailed binomial tests were computed using custom Python code. Furthermore, we analyzed success on the previously trained repetition trials to check for distraction or an overall lack of attentiveness to the testing situation in these test sessions.

## Results

Because the chimpanzees participated voluntarily, and the tasks became more difficult with time, our sample sizes declined progressively by attrition of participation during testing. Crucially, however, because the current study is focused on species capabilities, success by even a single individual suffices to demonstrate that some members of the species in question can perform the task. Two of the six chimpanzees trained in the feature-based dependency group successfully finished all training stages and continued with testing. Four chimpanzees of the arbitrary associative dependency group passed criterion in all training stages and were tested. Test results for all individuals are shown in Table 1. Performance in familiar repetition trials (that were differentially fed back and rewarded, and shown in between test trials) was used as a proxy for individuals’ attentiveness during test sessions: across a total of 34 test sessions (including all individuals and tests), repetition trials were mastered successfully in 30 sessions and failed in four instances. Below, if not indicated otherwise, familiar repetition trials were passed.

**Table 1:** Binomial test results for individual chimpanzees and tests

	1. Color	2. Extension	3. Novel shapes	4. Duplication	5. Position	6. CNE	7. CT	8. Edges	9. Inversion	10. Crossing
*Feature-based dependencies group*										
FK	*48/60* *<0.001*	*22/30* *0.01*	*24/30* *0.00*	*32/40* *<0.001*	*27/40* *0.02*	36/60 0.08	36/60 0.08			
KL	*50/60* *<* *0.001*	*21/30* *0.02*	*20/30* *0.05*	25/40 0.08	*26/40* *0.04*	33/60 0.26	36/60 0.08			
*Arbitrary association dependencies group*										
CI	*44/71* *0.03*	*20/30* *0.049*	*20/30* *0.049*	23/40 0.21	*27/40* *0.02*	24/47 0.50				
EV	*40/60* *0.01*	16/30 0.43	**19/30** **0.10**	**18/36** **0.57**						
PA	*37/60* *0.05*									
PE	*37/60* *0.05*	*21/30* *0.02*	19/30 0.10	16/40 0.92	*26/40* *0.04*	31/60 0.45		*29/40* *<0.001*	**18/30** **0.18**	*37/60* *0.05*

Numbers of correct or non-foil choices out of total number of trials above, with *P* values of binomial tests below. Significant test results are highlighted with italic in the respective cells. Bold cells indicate that the individual failed the familiar repetition trials presented during the test

Generally (a detailed presentation of the results is given below), all individuals tested (including both feature-based and arbitrary associative dependency groups) successfully generalized to colored stimuli (Test 1) after being trained with black shape stimuli only. All but one chimpanzee generalized the dependency rule over variable distances (Test 2), and three out of five individuals generalized to novel shape stimuli (Test 3). Both individuals trained for feature-based dependencies rejected stimuli lacking the dependency relation between the first and the last element. Unlike chimpanzees of the feature-based dependency group, individuals of the arbitrary dependency group did not have to generalize to novel-dependent elements, but had to ignore novel distractor elements. Only one chimpanzee of the arbitrary associative dependency group differentiated successfully between stimuli containing and lacking the dependency.

Further tests (Test 4–6) probed stimuli altering the dependencies in a specific way. Individuals across groups accepted stimuli with dependent elements at the edges of the visual patterns, while rejecting shifted dependency relations (Test 5). Most individuals accepted probes containing additional recurrences of dependent elements (Test 4 and 6).

### Feature-based dependency

Both individuals (FK and KL) in the feature-based dependency group successfully passed all generalization tests: they (i) generalized from black training stimuli to colored test stimuli, (ii) successfully completed the extension test (increasing stimulus length), and (iii) generalized the dependency rule to relationships between novel shapes (Tests 1, 2 and 3, see Table 1).

When tested with foils altering the AX*A rule in some way, both individuals rejected foils shifting the dependency position (Test 5), but accepted foils containing recurrences of dependent “A” elements in the center or near the edges of a visual pattern (Test 6). Individuals differed in how they treated recurrences of dependent elements at the edges of strings (Test 4).

Test 4 (Duplication Test) featured foils following an AAX*A or AX*AA pattern. While one individual (FK) rejected foil stimuli containing an additional recurrence of dependent elements at the edges of strings (32 first correct choices out of 40 test trials, *P* = 0.02), the other individual (KL) did not discriminate between stimuli with and without element duplication (25 correct out of 40, *P* = 0.08). Both individuals were above chance with familiar repetition trials presented during the duplication test (FK: 32 out of 42, *P* < 0.001; KL: 30 out of 42, *P* = 0.004), indicating good attentiveness during these sessions.

When tested for dependency position (Test 5), both individuals chose strings with dependent elements at the edges significantly more often than patterns with shifted dependency positions (FK: 27 out of 40, *P* = 0.02; KL: 26 out of 40, *P* = 0.04).

Finally, neither of the chimpanzees rejected foil strings in the last two tests. When tested with foil patterns containing recurrences of dependent elements in the center or near the edges of the string, both individuals treated foils and non-foils indiscriminately (FK: 36 out of 60, *P* = 0.08, KL: 33 out of 60, *P* = 0.26). Both were presented with 28 foils containing the recurrence in the center of the patterns (AX*AX*A) and 32 foils with recurrence of the first element near the edges of the stimulus (AXAX^2^A or AX^2^AXA). FK chose 18 (*P* = 0.09) and KL chose 16 (*P* = 0.29) non-foil strings out of a total of 28 trials presenting recurrences of dependent element in the center of the visual pattern. For foils with additional recurrences of dependent element near the edges, FK picked non-foil stimuli 18 times (*P* = 0.3), and KL did so 17 times (*P* = 0.43) out of 32 test trials. In both cases, the animals had statistically significant results for the familiar repetition trials shown in the same session (FK: 57/72, *P* < 0.01; KL: 59/66, *P* < 0.01).

Finally, neither of the two chimpanzees spontaneously transferred to a novel feature (color instead of shape) (FK: 36/60, *P* = 0.08; KL: 36/60, *P* = 0.08), but, once again, both were successful in the familiar repetition trials (FK: 53/60, *P* < 0.01; KL: 60/66, *P* < 0.01).

### Associative dependencies

Individuals in the associative dependencies group differed in their performance in the generalization tests: while all four subjects generalized to colored stimuli, and two out of three tested individuals generalized the AX*B rule over varying dependency distances, only one out of three tested chimpanzees rejected violations when novel distractor shapes were introduced (however, one of the two individuals who failed to generalize had below chance performance in familiar repetition trials).

Four chimpanzees successfully trained for arbitrary associative dependencies proceeded to Test 1 and generalized to colored stimuli (Table 1). Subject PA did not continue with further testing, thus three chimpanzees participated in the following probes: two generalization tests (“extension” and “novel shapes” test) and the first foil test (“duplication” test).

For the generalization tests, we found that individuals CI (20/30, *P* < 0.05) and PE (21/30, *P* = 0.02) applied the dependency rule to a varying dependency distance (Test 2). Although CI successfully generalized over dependency distance, she failed in familiar repetition trials (17/28, *P* = 0.173). Individual EV did not differentiate between patterns that did and did not contain the dependent items when stimulus length was increased (16/30 test trials, *P* = 0.43; training trials: 26/36, *P* = 0.01). When novel distractor shapes (Test 3) were introduced between dependent items, only CI (20/30, *P* < 0.05) preferentially chose patterns containing the trained associative pairs. Neither EV (19/30, *P* = 0.10) nor PE (19/30, *P* = 0.10; repetition trials 22/30, *P* = 0.01) rejected strings lacking the dependency when patterns included novel distractor shape elements. However, EV’s unsuccessful choices in familiar repetition trials suggested a general lack of attentiveness (17/29, *P* = 0.23).

In the course of the foil tests, sample size decreased successively. None of the three chimpanzees rejected foils with recurrences of dependent elements at the edges of strings (Test 4). Two subjects tested with dependent element recurrences in the center or near the edges of visual patterns (Test 6) correctly differentiated between foils and non-foils. All individuals tested with shifted dependency positions (Test 5, two individuals tested) correctly rejected foil stimuli. One individual was presented with stimuli missing elements at the edges of strings (Test 8), inverted (B before A, Test 9), and scrambled (AiX*Bj, Test 10) dependency stimuli. She rejected foils in all tests except in Test 9 (where she also failed familiar repetition trials).

In Test 4, none of the three individuals rejected duplication foils (CI: 23/40, *P* = 0.21; EV: 18/36, *P* = 0.57; PE: 16/40, *P* = 0.92). CI (32/42, *P* < 0.01) and PE (31/42, *P* < 0.01) succeeded at familiar repetition trials in that test.

Two individuals were tested further (CI and PE). Both chimpanzees were significantly more likely to choose strings with dependent elements at the edges over foils with shifted dependency positions (Test 5) (CI: 27/40, *P* = 0.02; PE: 26/40, *P* = 0.04).

In Test 6, foils containing recurrences of either As or Bs in the center or near the edges of the visual patterns were accepted by both subjects (but CI did not complete all trials of the test: 24/47, *P* = 0.5; PE; 31/60, *P* = 0.45). CI chose the non-foil stimulus 13 out of 19 times when presented with recurrences in the center (*P* = 0.08), but only 11 out of 28 times when presented with recurrences near the edges of the patterns (*P* = 0.91). PE had similar results for both center and near-edge foil patterns (center: 13/25, *P* = 0.5; near edges: 18/35, *P* = 0.5). Choices in familiar repetition trials indicated good attentiveness during the test for both individuals (CI: 31/48, *P* = 0.03; PE: 48/60, *P* < 0.01).

Only one female (PE) underwent and completed the last three tests. Strings missing either an A or a B at the edges of patterns (Test 8) were rejected (29/40, *P* < 0.001). However, a closer examination showed that PE rejected foil stimuli with the first element missing (18/21, *P* < 0.01) but treated foils lacking the last dependency element as acceptable (11/19, *P* = 0.32), suggesting an “initial” or left-edge bias. When A’s were placed at the end of the stimulus and B’s at the beginning (Test 9), this subject chose randomly between foil and non-foil patterns (18/30, *P* = 0.18), but also failed in the familiar repetition trials (19/30, *P* = 0.1) suggesting distraction or a lack of attentiveness during this test. In the final test (Test 10) where the associative pairs were scrambled (A_*i*_ was presented with B_*j*_ at the edges of the stimuli), PE differentiated between foils and scrambled associative pairs (37/60, *P* < 0.05), potentially indicating some generalization over the arbitrary associative item categories.

## Discussion

Overall, our results demonstrate success and considerable flexibility in extracting and generalizing dependency regularities in artificial, abstract, visual patterns by chimpanzees. Our study subjects successfully formed identity relations between elements based on the common shared feature “shape”, and also based on learned associative pairings of specific items. Generalization tests demonstrated that chimpanzees matched elements independently of additional distracting information (color) across varying distances and to novel items or item combinations. Similarly monkeys are able to ignore distracting information (such as color, shape, or surface area) in number matching experiments (Cantlon and Brannon 2007; Jordan et al. 2008), and can even match number information across sensory modalities (Jordan et al. 2008).

When computing *feature*-*based dependencies*, two different items have to be identified as sharing some feature. Spatial or temporal relationships between linked individual elements could then be further processed and computed (Marcus et al. 1999). Our chimpanzees proved capable of generalizing to novel shapes: both individuals of the AA group applied this dependency rule to novel arbitrary shapes, and did so regardless of dependency distance. Stimuli could not be discriminated on the basis of color information or length alone. Moreover, as any arbitrary shape could become a dependency element, individuals could not decide on the correctness of a pattern by looking merely at either the first or the last element. That is, individuals could not have differentiated between AX*A and AX* strings without matching the dependent elements. This finding supports and expands previous results in squirrel monkeys, who matched same class acoustic elements located at the beginnings and ends of strings (Ravignani et al. 2013b). While the elements for the squirrel monkeys were drawn from two perceptually distinct categories (high- or low-pitched sounds), chimpanzees in our study were able to process a more abstract and multi-dimensional relational dependency (same or different shape). Crucially, both AA-subjects generalized to stimuli containing entirely unfamiliar elements (novel shapes and novel colors). This stands in contrast to results in various bird species that have shown comparatively limited generalization capabilities (van Heijningen et al. 2009, 2013; Stobbe et al. 2012; ten Cate and Okanoya 2012). Our results show that chimpanzees are capable of representing a same feature dependency rule and also to perform some location-based computations with them.

Both individuals of the AA group were tested for their ability to spontaneously transfer the dependency regularity to a novel feature (Test 7), while training stimuli contained dependencies between shapes, and this test probed whether chimpanzees would spontaneously extend the “same edges” rule to dependencies between items with the same color but different shapes. While neither generalized to this novel category, the results can presumably be simply attributed to training circumstances, because both individuals learned to ignore color as distracting information in the previous six test stages. In the future, further tests presenting entirely novel categories (e.g., based on size, orientation, etc.) could shed more light on chimpanzees’ transfer capabilities and consequently on the level of encoding.

Recognizing the presence of specific items is not sufficient to process arbitrary associative dependencies. Instead, relations between two elements have to be established by repeatedly experiencing their co-occurrence and thereby learning their association (i.e., A always appears with B). In our experiment, because elements defining arbitrary associative dependencies did not share any non-arbitrary feature, generalization to novel, unfamiliar items could not be tested in the AB group. However, when multiple pairs with the same item relations were learned, individual elements of associative pairs could be rearranged. The only individual who successfully completed all tests (PE) did accept novel combinations of prefix (any A) and suffix (any B) items (Test 10), suggesting that this subject formed a category-wide precedence rule. Arguably, the individual could have based her choices on the presence or the absence of the first or the last element only, which would have allowed for significant results in the “crossing” test (Test 10) without noticing the recombination of trained item pairs. Looking at side biases in the “position” and the “edges” test renders this unlikely however; PE did show a left-edge bias (first element missing: 18/21, *P* < 0.01; last element missing: 11/19, *P* = 0.32) in the “edges test” (Test 8; lacking one dependent element either at the beginning or at the end of the visual pattern), but a right-edge bias (first element shifted inwards: 11/20, *P* = 0.42; last element shifted inwards: 15/20, *P* = 0.02) in the “position test” (Test 5; one of the dependent elements shifted inwards). Thus, the individual seems to be sensitive to both stimulus edges. But positive results in the crossing test (Test 10) have to be contrasted with results in the inversion test (BXA, Test 9) to show category-wide generalization. Due to a lack of task attentiveness during the inversion task (based on failed familiar trials), we do not attempt to draw conclusive inferences from this test.

All further tests presented foils altering, but not violating, the dependency rule of the trained visual patterns. One male (FK) of the abstract dependency group rejected patterns where the first or the last dependency element was duplicated (duplication test, AAX*A, AX*AA). Three other individuals (one from the AA group and two of the AB group) did not differentiate between foils and S+ strings. This result strongly suggests that FK (AA group) not only matched the first and the last elements of a string, but also paid attention to the second and next to last elements. He seemed to be unique in that respect, as the test results of the other three individuals suggest that they ignored internal elements. FK’s differentiation is particularly noteworthy, as he developed it spontaneously: no previous training step or test required attention to other elements besides the first and the last. This was different in Test 5, where the relative position of the dependency was tested. The dependent elements were contained in the strings, but their positions were shifted. The foil strings followed an XAX*A or AX*AX and an XAX*B or AX*BX pattern, respectively. As in the training trials, the stimulus feature that was being tested was located at the edges of the stimulus, and all four individuals rejected these foils. Looking at performance for center or near-edge foils (Test 6), we found further support for a bias to focus on stimulus edges. None of the four chimpanzees rejected foils containing a center or near-edge additional recurrence of a dependency element.

In sum, our results show that one crucial requirement for processing regularities, namely the ability to understand and represent relationships between elements separated in space and time (Gebhart et al. 2009; Newport and Aslin 2004; van Heugten and Shi 2010), is also present in one of humans’ closest living relative, the chimpanzee. The ability to form abstractions and rules, and flexibly apply these rules to novel stimuli, is essential for such regularity computations.

Given that humans are not the only species capable of such computations, we may ask what selective value they possess in the absence of language or music. Similar instances of flexible dependency processing have been demonstrated for computing and representing social information, or when planning or observing motor actions (Bergman et al. 2003; Emery and Clayton 2004; Maclean et al. 2008; Wittig et al. 2014; Wolpert et al. 2001, 2003), and analogies between the computational processes underlying motor actions, action observation, and social cognition have been suggested (Wolpert et al. 2001, 2003). Understanding relationships between phenomena that are separated in space and time is a fundamental cognitive ability that is valuable when processing social relationships between group members or understanding connections between distant elements in an action chain (e.g., during tool use; McGrew 2004; Sanz et al. 2004).

Alternatively, recent results using relatively arbitrary auditory and visual stimuli might suggest that encoding regularities allowing an organism to process relevant structures might take place at an abstract level that applies across multiple cognitive domains. Such multi-domain capabilities may have been employed by the chimpanzees in our study when learning feature based and arbitrary associative dependency regularities in visual patterns. We hope that future research testing and connecting regularity processing abilities in the general sensory, social, and technological domains will help shed further light on this topic.

## Electronic supplementary material

Below is the link to the electronic supplementary material.

Supplementary material 1 (DOC 45 kb)

Supplementary material 2 (TIFF 244 kb)

Supplementary material 3 (TIFF 36 kb)

Supplementary material 4 (TIFF 63 kb)

Supplementary material 5 (TIFF 183 kb)
