# Supplementary material for: Non-adjacent visual dependency learning in chimpanzees
Source: Anim Cogn. 2015 Jan 21;18(3):733–45. doi: 10.1007/s10071-015-0840-x (PMC4412729; doi:10.1007/s10071-015-0840-x)
Supplement: Supplementary file 1 — Supplementary material 1 (DOC 45 kb) [file 10071_2015_840_MOESM1_ESM.doc]

**Supplementary Material**

Annex A: *Experimental Area in Budongo Trail*

Budongo Trail allows for strictly non-invasive research. Individuals were participating on an entirely voluntary basis, and could leave the experiments at any time. The experiments described below were conducted in an off-show area of the chimpanzee enclosure, located in between two indoor pods. Individuals could access the experimental area from two sides on two levels (tunnel system above the experimental area and entrance from the pods). The experimental area was divided into 5 chimpanzee compartments adjacent to each other and a “researcher aisle”, which was separated from the chimpanzee area by a wire mesh (Figure A1). Chimpanzees could participate to trainings and experiments in any of the 5 compartments. All compartments were open and accessible at all times.

Fig A1

Annex B: *Description of training units per group*

*1. Training for the AAgroup:*

Individuals had to pass (criterion was set to 33 first correct choices within a total of 48 trials; power analysis using a binomial distribution, *P*<0.001) two training units before they were tested:

1. Two-element stimuli (geometrical shapes in black on transparent background with black frames, Figure A2) were presented. Individuals were rewarded for choosing stimuli with homogenous elements (AA) over heterogeneous ones (AX) in a 2AFC-task.

Fig A2

1. Individuals who successfully finished the first training unit continued with the second training stage where one or two “distractor” elements were introduced in between dependency elements (AXA and AXXA) (Figure A3).

Fig A3

Two individuals successfully completed these training units and were tested consequently.

*2. Training for the ABgroup:*

1. In a first training step individuals were trained to choose any two-element heterogeneous (AB) stimuli over homogenous (AA) ones (see Figure A4.1).
2. Subsequently individuals were presented with a set of five specific associative pairs of heterogeneous elements (A_1_B_1_, A_2_B_2_, A_3_B_3_, A_4_B_4_, and A_5_B_5_). These had to be chosen over any other heterogeneous element pair (XY, sampled from another pool of 45 different shapes). Those were perceptually different (numbers and letters drawn in fine line) from the AB-associative pair elements (Figure A4.3).
3. For the next training step AB-pairs were presented with heterogeneous element stimuli that were perceptually similar (distractor shapes) to the ones of the AB-pairs (see Figure A4.4).
4. In the final training unit (Figure A4.4), one or two “distractor” elements (X’s) were inserted between associative pairs, producing stimuli such as A_i_XB_i_ and A_i_XX’B_i_ (where X≠X’). These positive stimuli were presented together with negative ones composed of three or four heterogeneous elements (XXX, XXXX).

Fig A4

**Figure Captions**

**Fig A1**: The testing area. The chimpanzees were tested in an off-show area located in the middle of two indoor pods (2 and 3). Individuals could access this area on the lower level from two sides (Pod 2 and Pod 3) as well as from two sides on an upper level (tunnel system linking Pod 1 and Pod 2). The chimpanzee testing area was divided in five individual compartments, which were never shut during the experiments (individuals were not separated from the group). A wire mesh divided the chimpanzee area from the experimenter area. The touch screen setup (touch screen, computer, loud speakers) was mounted on a movable table. Thus the experimenter could position the setup flexibly in front of individual chimpanzees in any of the five chimpanzee compartments. A keeper was present at all times during the experiments performing husbandry training. This guaranteed that an individual taking part in the experiment would not be disturbed by others interfering.

**Fig A2**: Example of a training stimulus. The chimpanzees were trained to choose the stimulus containing identical elements (a) over the stimulus with heterogeneous elements (b).

**Fig A3**: Example of stimuli in training unit 2. The sequence of pictures follows an AX^n^A pattern, where n=1, or 2.

**Fig A4**: Training stages for the AB group. The right column shows examples of rewarded stimuli (S+), the left column depicts examples of negative stimuli (S-). In a first training stage (1) any pair of heterogeneous elements had to be chosen over homogenous ones. In a second training stage (2), S+ and S- stimuli consisted of heterogeneous elements, where S+ was any of the associative pairs (A_i_B_i_) and S- consisted of two different elements sampled from a pool of numbers and letters (perceptually markedly different to S+ stimuli). In the third training step (3) 2-element S+ and S- stimuli that were perceptually similar were presented. Any of the five associative pairs (S+) was presented with two heterogeneous shapes (S-). In the final training stage (4) one or two distractor elements were inserted in between associative pair elements (A_i_XA_j_ and A_i_XXA_j_) and presented simultaneously with stimuli consisting of three or four heterogeneous elements.
