# Supplementary figures and images for: Non-adjacent visual dependency learning in chimpanzees
Source: Anim Cogn. 2015 Jan 21;18(3):733–45. doi: 10.1007/s10071-015-0840-x (PMC4412729; doi:10.1007/s10071-015-0840-x)

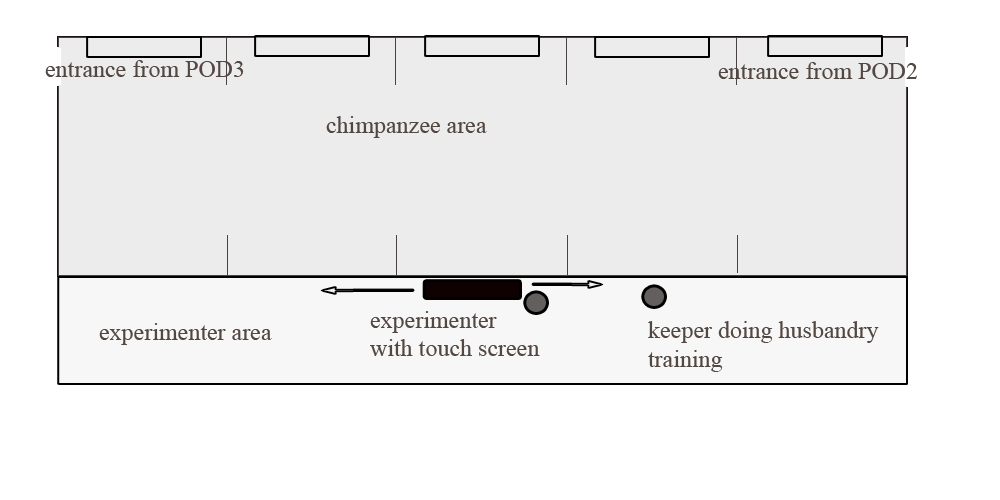

Supplement: Supplementary file 2 — Supplementary material 2 (TIFF 244 kb) [file 10071_2015_840_MOESM2_ESM.tif]

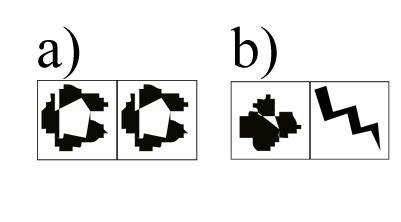

Supplement: Supplementary file 3 — Supplementary material 3 (TIFF 36 kb) [file 10071_2015_840_MOESM3_ESM.tif]

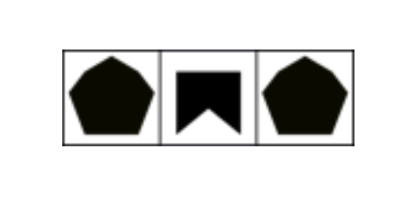

Supplement: Supplementary file 4 — Supplementary material 4 (TIFF 63 kb) [file 10071_2015_840_MOESM4_ESM.tif]

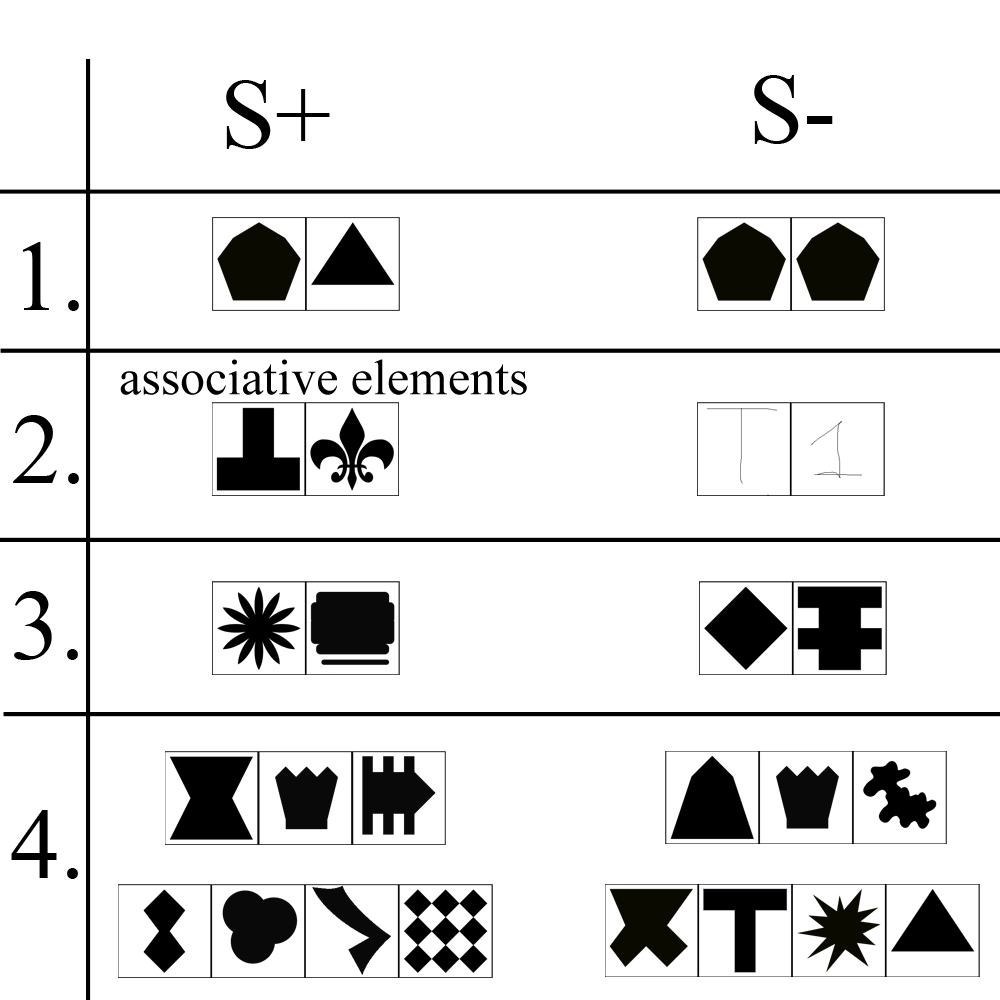

Supplement: Supplementary file 5 — Supplementary material 5 (TIFF 183 kb) [file 10071_2015_840_MOESM5_ESM.tiff]
